# Empagliflozin ameliorates renal and metabolic derangements in obese type 2 diabetic mice by blocking advanced glycation end product–receptor axis

**DOI:** 10.1186/s10020-025-01138-0

**Published:** 2025-03-06

**Authors:** Takanori Matsui, Ami Sotokawauchi, Yuri Nishino, Yoshinori Koga, Sho-ichi Yamagishi

**Affiliations:** 1 Department of Bioscience and Biotechnology, Fukui Prefectural University, Eiheiji, 910-1195 Japan; 2 Department of Pediatric Surgery, Kurume University School of Medicine, Kurume, 830-0011 Japan; 3 Department of Medicine, Division of Nephrology, Kurume University School of Medicine, Kurume, 830-0011 Japan; 4 Department of Medicine, Division of Diabetes, Metabolism, and Endocrinology, Showa University Graduate School of Medicine, Tokyo, 142-8666 Japan

**Keywords:** AGEs, Empagliflozin, Oxidative stress, RAGE, Renal and metabolic disease

## Abstract

**Background:**

Although randomized clinical trials revealed that inhibitors of sodium-glucose cotransporter 2 (SGLT2) reduced the risk of cardiovascular and renal events in patients with type 2 diabetes, the underlying molecular mechanisms remain to be elucidated. Since there is accumulating evidence that AGEs and their receptor (RAGE) play a crucial role in diabetes-related complications, we examined here whether empagliflozin ameliorates renal and metabolic derangements in db/db mice, an obese type 2 diabetes animal by blocking the AGE-RAGE axis.

**Methods:**

Eight-week-old db/db mice were fed a 0.045% empagliflozin diet (db/db + Empa) or normal diet (db/db) for 13 weeks. The same week-old db/ + m mice were maintained on normal diet (db/ + m) used as a control. At baseline and 13 weeks after intervention, biochemical analyses in the serum and urine were performed, and kidneys and adipose tissues were obtained for morphological, immunohistochemical, and reverse transcription-polymerase chain reaction analyses.

**Results:**

Empagliflozin treatment for 13 weeks significantly reduced AGEs, *N*^ε^-(5-hydro-5-methyl-4-imidazolon-2-yl)-ornithine (MG-H1), RAGE, NADPH oxidase-derived oxidative stress, inflammatory and fibrotic reactions in the kidneys of db/db mice of 21-week-old in association with attenuation of glomerular extracellular matrix accumulation, podocyte loss, proteinuria, and tubulointerstitial damage. Empagliflozin also reduced the AGE-RAGE-oxidative stress-induced inflammatory reactions in the adipose tissues of db/db mice, which was associated with restoration of adiponectin levels and decreased insulin resistance. Serum MG-H1 levels of control and db/db mice at 21 weeks of age were significantly associated with proteinuria, tubulointerstitial damage, tissue AGEs levels, and serum monocyte chemoattractant protein-1 and adiponectin (inversely) values.

**Conclusions:**

Our present findings suggest that empagliflozin could ameliorate renal and metabolic derangements in type 2 diabetes animals by attenuating the AGE-RAGE axis.

## Introduction

According to the report of Diabetes Atlas 10th Edition 2021, 537 million adults are estimated to be living with diabetes, and the number of patients with diabetes is predicted to increase to 734 million by 2045 (Ogurtsova et al. 2022). The rise in number of patients with type 2 diabetes mellitus is due to aging of the world population, unhealthy life habits, and increasing prevalence of obesity, which in concert could cause insulin resistance and eventually chronic hyperglycemia (Ogurtsova et al. 2022; Barbieri et al. 2024). The risk of cardiovascular and renal events is dramatically increased in type 2 diabetes patients; half of them die from these devastaing disorders, and more than 10% of the global health expenditure (966 billion US dollars) is spent for the treatment of diabetes (Ogurtsova et al. 2022; Rao Kondapally Seshasai et al. 2011). These observations suggest that prevention and management of obesity, insulin resistance and its-associated cariovascular and renal disease are of utmost importance in patients with type 2 diabetes.

Inhibitors of sodium-glucose cotransporter 2 (SGLT2is) are an oral hypoglycemic agent, which ameliorates hyperglycemia in type 2 diabetes by promoting urinary glucose excretion via suppression of glucose reabsorption in S1 segment of renal proximal tubule (Yanagisawa et al. 2020). Systematic review and meta-analyses of randomized clinical trials have shown that SGLT2is significantly reduce the risk of all-cause death, major cardiovascular events, and renal composite outcome in patients with type 2 diabetes (Marilly et al. 2022; Yang et al. 2024). Since SGLT2i has also been reported to reduce body weight, waist circumference, and visceral fat volume in patients with type 2 diabetes (Bekki et al. 2019), SGLT2is could be a promising oral hypoglycemic agent for the treatment of type 2 diabetes with obesity and insulin resistance, especially those with a previously history of cardiovascular disease. However, the underlying molecular mechanisms for cardiorenal and metabolic protection by SGLT2is are not fully understood.

Monosaccharide, such as glucose, fructose, and glyceraldehyde react non-enzymatically with amino groups of proteins, nucleic acids, and lipids to form Amadori products and then by a complex series of rearrangement, dehydration, and condensation to generate senescent pathological macromolecules called advanced glycation end products (AGEs) over a course of days to weeks (Yamagishi and Matsui 2019). The formation and accumulation of AGEs has progressed under a physiological aging process, especially under chronic hyperglycemic and inflammatory conditions, and engagement of the receptor for AGEs (RAGE) with AGEs elicits oxidative stress generation and evokes inflammatory reactions, thereby being involved in various aging-related cardiorenal and metabolic disorders (Yamagishi and Matsui 2019; Ramasamy et al. 2011; Genuth et al. 2005; Thomas et al. 2005; Yamagishi 2012). Several experimental studies have suggested that SGLT2is could exhibit anti-aging effects by improving many of aging-related pathological processes (Russo et al. 2024). Given the active involvement of AGE-RAGE axis in numerous kinds of aging-related complications in diabetes (Yamagishi and Matsui 2019; Ramasamy et al. 2011; Genuth et al. 2005; Thomas et al. 2005; Yamagishi 2012), SGLT2is may work as anti-AGEs agents. However, the role of pharmacological interventions with SGLT2is in the AGE-RAGE signaling pathway, especially in diabetes-associated cardiorenal and metabolic disorders remains unknown. Therefore, we examined here whether empagliflozin ameliorates renal and metabolic derangements in db/db mice, an obese type 2 diabetes animal by blocking the AGE-RAGE axis.

## Methods

### Materials

Animal diets with or without 0.045% empagliflozin were generously provided by Boehringer Ingelheim (Ingelheim, Germany). *N*^ε^-(5-hydro-5-methyl-4-imidazolon-2-yl)-ornithine (MG-H1) acetate salt was purchased from Iris Biotech (Marktredwitz, Germany).

### Animals

Male db/db mice (BKS.Cg- + Lepr^db^/ + Lepr^db^/Jcl) and db/ + m mice (BKS.Cg-m + / + Lepr^db^/Jcl) were purchased from CLEA Japan (Tokyo, Japan). Eight-week-old db/db mice were fed a 0.045% empagliflozin diet (db/db + Empa) or normal diet (db/db) for 13 weeks randomly. The same week-old db/ + m mice were maintained on normal diet (db/ + m) used as a control.

Blood pressure and heart rate of 8- and 21-week-old mice were measured as described previously (Matsui et al. 2017). Hematocrit was measured using an automated hematology analyzer KX-21 (Sysmex, Kobe, Japan). Echocardiography of 21-week-old mice was performed under isoflurane anesthesia using Vevo 3100 Imaging System (FujiFilm VisualSonics, Toronto, Canada), and the E/eʹ ratio (mitral inflow velocity/tissue Doppler mitral annulus velocity) was calculated using the Vevo LAB version 2.1.0 software (FujiFilm VisualSonics). Then mice were sacrificed after an overnight fasting, blood, kidneys, and adipose tissues were obtained for NADPH oxidase activity assay, real-time reverse transcription-PCR (RT-PCR), biochemical, immunohistochemical and morphological analyses.

Serum C-reactive protein (CRP), insulin, and adiponectin and MCP-1 were measured with ELISA systems from R&D Systems (Minneapolis, MN), Shibayagi (Gunma, Japan), and Abcam (Cambridge, UK), respectively. HOMA-IR was calculated according to the following formula: HOMA-IR = blood glucose (mg/dL) × insulin (μU/mL)/405. Other blood chemistry was analyzed with standard enzymatic methods as described previously (Matsui et al. 2017). All the data were included and analyzed in a blind manner. All experimental procedures were conducted in accordance with the National Institutes Health Guide for Care and Use of Laboratory Animals and were approved by the ethnical committee of Kurume University School of Medicine (Approval No. 2019-160, 2020-092, 2021-082).

### Measurement of urinary protein and kidney injury molecular-1 (KIM-1) excretions, and *N*-acetyl-β-d-glucosaminidase (NAG) activity

Proteinuria was determined with Protein Assay BCA kit from Nacalai Tesque (Kyoto, Japan) as described previously (Beretov et al. 2014). Urinary KIM-1 excretion was measured with ELISA (R&D Systems). Urinary NAG activity was measured by a colorimetric assay kit BioVision (Milpitas, CA). Urinary creatinine levels were measured using a Serotec CRE-L (Serotec, Sapporo, Japan).

### ELISA for MG-H1

Serum MG-H1 levels were measured with ELISA. In brief, 96-well plate was coated with 1 µg/mL MG-H1-conjugate BSA, and then twofold diluted mice serum (50 µL) were added to each well as a competitor against 50 µL of 1:5000-diluted horseradish peroxidase-labeled MG-H1 antibody (STA-011; Cell Biolabs, San Diego, CA, USA). After 2 h-incubation at 4 °C, colorimetric substrate was added to the well. Absorbance at 450 nm was measured. Serum protein levels were measured with Pierce 660 nm Protein Assay Kit (Thermo Fisher Scientific K.K., Tokyo, Japan).

### Immunostaining and morphological analysis

The kidney and adipose tissue sections were incubated overnight at 4 °C with antibodies, and the reactions were visualized with a Histofine Simple Stain Rat MAX-POMULTI kit (Nichirei Co., Japan) as described previously (Matsui et al. 2017). Antibodies raised against AGEs, MG-H1 (Cell Biolabs), RAGE (SC-5563; Santa Cruz Biotechnology, Dallas, TX), F4/80 (SC-25830; Santa Cruz Biotechnology), nitrotyrosine (SMC-154D; StressMarq Biosciences, British Columbia, Canada), MCP-1 (ab7202; Abcam), type I collagen (ab34710; Abcam), podocin (ab181143; Abcam), KIM-1 (ab47635; Abcam), tumor necrosis factor-α (TNF-α) (ab34674; Abcam), Wilms' tumor 1 (WT1) protein (M3561; Dako, Santa Clara, CA, USA), 4-hydroxynonenal (4-HNE) (MHN-020P; NIKKEN SEIL, Shizuoka, Japan), adiponectin (SC-53910; Santa Cruz Biotechnology), and plasminogen activator inhibitor-1 (PAI-1) (SC-8979; Santa Cruz Biotechnology) were used for immunostaining and western blot analyses. Immunohistoreactivity in 5 different fields of each sample was quantified by percentage and intensity of brown stainings, and 3 µm paraffin sections were stained with Masson’s trichrome for light microscopic analysis (Matsui et al. 2017). Fold respected to 8-week-old control mice.

### RT-PCR

Real-time RT-PCR was performed as described previously (Matsui et al. 2017). Identifications of primers for mouse *Rage*, *p22phox*, NADPH oxidase 1 (*Nox1*), NADPH oxidase 2 (*Nox2*, also known as *gp91phox*), NADPH oxidase 4 (*Nox4*), *p47phox*, *p67phox*, *Mcp-1*, type I collagen, *Kim-1*, *Tnf-α*, *adiponectin*, and 18S genes were Mm00545815_m1, Mm00514478_m1, Mm00549170_m1, Mm01287743_m1, Mm00479246_m1, Mm00447921_m1, Mm00726636_s1, Mm00441242_m1, Mm00801666_g1, Mm00506686_m1, Mm00443258_m1, Mm00456425_m1, and Mm03928990_g1, respectively. Data were normalized by the intensity of 18s-derived signals and then related to the value of 8-week-old db/ + m mice.

### Measurement of NADPH oxidase activity

NADPH oxidase activity was measured by a luminescence assay (Matsui et al. 2017).

### Statistical analysis

All values were presented as mean ± SD. Statistical analysis was conducted using R version 4.3.1 software (The R Foundation for Statistical Computing Platform, Vienna, Austria). Data normality was tested using the Shapiro–Wilk normality test. The normally distributed data were followed by ANOVA with the Tukey–Kramer test. For data that not follow a normal distribution, Steel–Dwass test was performed. The parametric coefficient of Pearson’s correlation was used to analyze the correlation between the two groups. All p-value < 0.05 was considered significant.

## Results

### Clinical characteristics of animals

Clinical characteristics of animals are shown in Table 1. Body weight, food intake, fasting blood glucose, HOMA-IR, total-cholesterol, HDL-cholesterol, blood urea nitrogen, CRP, AST, ALT, and ratio of liver weight to femur length in 8-week-old db/db diabetic mice were significantly higher than those of non-diabetic db/ + m mice of the same age. Furthermore, compared with 21-week-old db/ + m mice, body weight, food intake, fasting blood glucose, HOMA-IR, total-cholesterol, HDL-cholesterol, CRP, serum MG-H1, ALT, and ratios of kidney and liver weights to femur length, and E/eʹ ratio were significantly elevated in 21-week-old db/db diabetic mice (Table 1). Thirteen-week empagliflozin treatment significantly inhibited the increase in fasting blood glucose, HOMA-IR, CRP, serum MG-H1, ratio of liver weight to femur length, and E/eʹ ratio in 21-week-old db/db diabetic mice, while body weight, food intake, and blood urea nitrogen of empagliflozin-treated diabetic mice at 21 weeks of age were higher than those of non-treated diabetic mice of the same age.

**Table 1:** Clinical characteristics of mice

Characteristics	8-week-old mice		21-week-old mice		
	db/ + m	db/db	db/ + m	db/db	db/db + Empa
Number	5	5	6	6	6
Body weight (g)	24.6 ± 1.0	36.1 ± 0.5**	32.7 ± 0.9	47.2 ± 5.3††	57.5 ± 3.6‡‡
Food intake (g/day)	4.0 ± 0.2	8.5 ± 0.2**	2.8 ± 0.4	4.9 ± 0.2††	6.6 ± 0.0‡‡
Heart rate (beats/min)	508 ± 78	532 ± 42	594 ± 64	541 ± 30	563 ± 53
Mean blood pressure (mmHg)	92 ± 5	90 ± 7	97 ± 6	89 ± 7	92 ± 7
Fasting blood glucose (mg/dL)	139 ± 17	296 ± 60**	103 ± 21	601 ± 68††	173 ± 26‡‡
Hematocrit (%)	48 ± 6	54 ± 8	46 ± 2	46 ± 6	49 ± 3
HOMA-IR	16 ± 8	86 ± 20**	5 ± 5	168 ± 32††	71 ± 14‡‡
Total-cholesterol (mg/dL)	67 ± 5	96 ± 7**	72 ± 6	108 ± 20††	127 ± 17
HDL-cholesterol (mg/dL)	56 ± 5	97 ± 7**	68 ± 6	119 ± 26††	148 ± 22
Triglycerides (mg/dL)	131 ± 21	46 ± 12	62 ± 40	118 ± 64	48 ± 23
Blood urea nitrogen (mg/dL)	19 ± 4	27 ± 3**	20 ± 5	21 ± 4	29 ± 6‡
Creatinine (mg/dL)	0.17 ± 0.03	0.21 ± 0.05	0.25 ± 0.17	0.18 ± 0.04	0.18 ± 0.07
Kidney weight/femur length(mg/mm)	14 ± 2	15 ± 2	16 ± 2	22 ± 3††	21 ± 3
Serum CRP (μg/mL)	9.5 ± 0.3	16.8 ± 4.7**	8.0 ± 1.9	18.4 ± 2.4†	11.5 ± 1.6‡
Serum MG-H1 (ng/mg protein)	23.8 ± 5.0	16.1 ± 3.6	14.9 ± 2.6	29.1 ± 8.2††	17.9 ± 4.7‡
AST (U/L)	78 ± 49	273 ± 177**	305 ± 317	254 ± 76	316 ± 245
ALT (U/L)	65 ± 44	235 ± 233**	80 ± 55	224 ± 91††	220 ± 127
Liver weight/femur length (mg/mm)	86 ± 6	183 ± 23**	91 ± 15	230 ± 21††	185 ± 28‡
Heart weight/femur length (mg/mm)	10 ± 2	10 ± 1	12 ± 1	12 ± 1	12 ± 1
E/e’	–	–	19.6 ± 7.2	33.6 ± 4.4††	20.3 ± 6.5‡

Data are presented as mean ± SD

^**^, p < 0.01 compared with 8-week-old db/ + m mice. † and ††, p < 0.05 and p < 0.01 compared with 21-week-old db/ + m mice, respectively. ‡ and ‡‡, p < 0.05 and p < 0.01 compared with 21-week-old db/db mice, respectively

### Effects of empagliflozin on AGE-RAGE-oxidative stress axis in the kidneys of diabetic mice

**Fig. 1:**
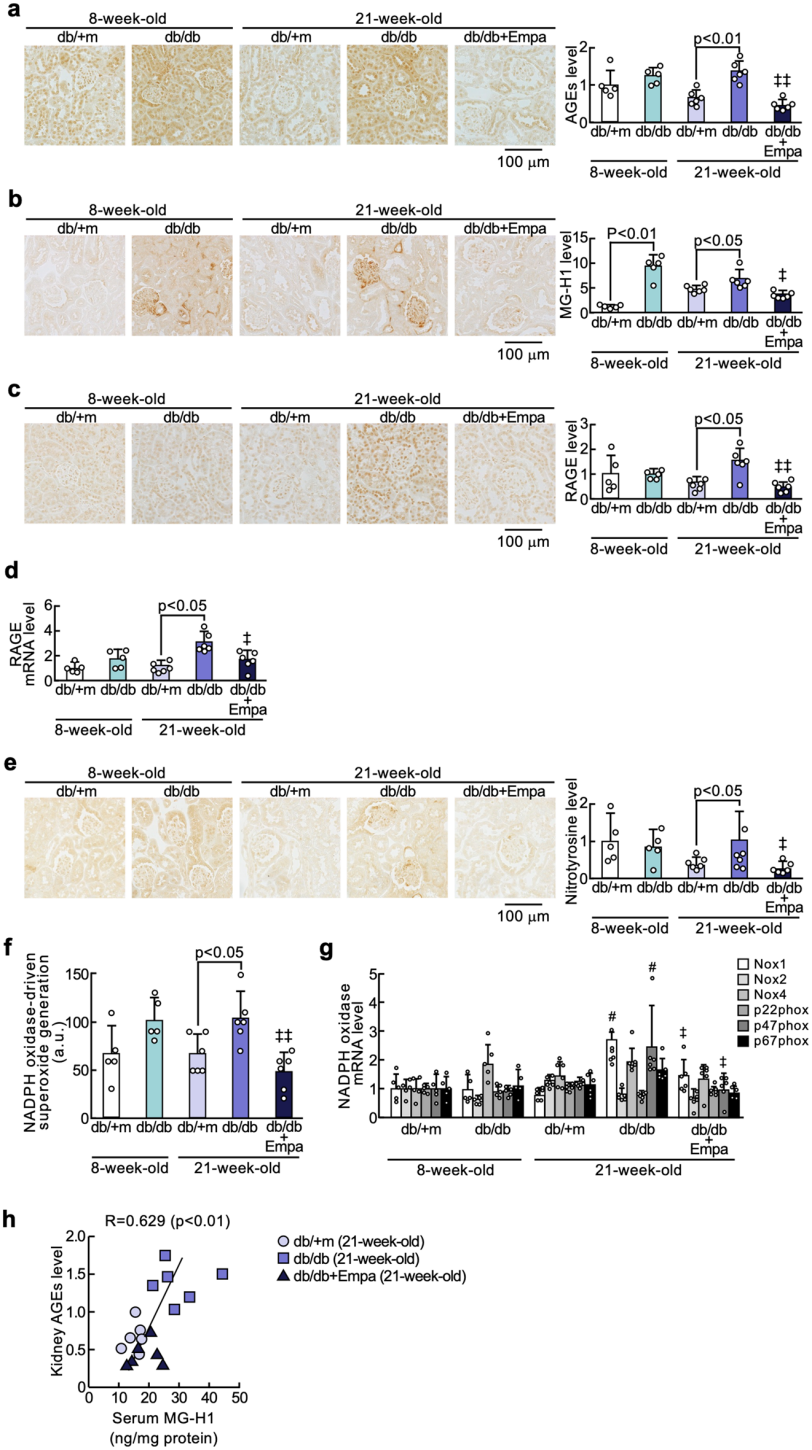
Effects of empagliflozin on AGE-RAGE-oxidative stress axis in the kidneys of diabetic mice. Levels of AGEs (**a**), MG-H1 (**b**), RAGE protein (**c**), RAGE mRNA (**d**), nitrotyrosine (**e**), NADPH oxidase-derived superoxide generation (**f**), and gene expression levels of components of NADPH oxidase (**g**). **a–c** and **e**: Each left panel shows representative immunostainings of AGEs (**a**), MG-H1 (**b**), RAGE protein (**c**), and nitrotyrosine (**e**) in the kidneys. Each right panel shows the quantitative data. **h**: Correlation of serum MG-H1 with kidney AGEs levels. #, p < 0.05 compared with 21-week-old non-diabetic db/m + mice. ‡ and ‡‡, p < 0.05 and p < 0.01 compared with 21-week-old diabetic db/db mice, respectively

As shown in Fig. 1a–g, although there were no significant differences in markers of AGE-RAGE axis except for MG-H1 levels between diabetic and non-diabetic mice at 8 weeks of age, AGEs, MG-H1, RAGE mRNA and protein levels, nitrotyrosine, an oxidative stress marker, NADPH oxidase-derived superoxide generation, and gene expression levels of components of NADPH oxidase, such as *Nox1* and *p47phox* were significantly increased in the kidneys of 21-week-old diabetic mice, all of which were inhibited by empagliflozin treatment for 13 weeks. Serum MG-H1 levels of mice at 21 weeks of age were highly correlated with AGEs levels in the kidneys (Fig. 1h).

### Effects of empagliflozin on renal damage of diabetic mice

**Fig. 2:**
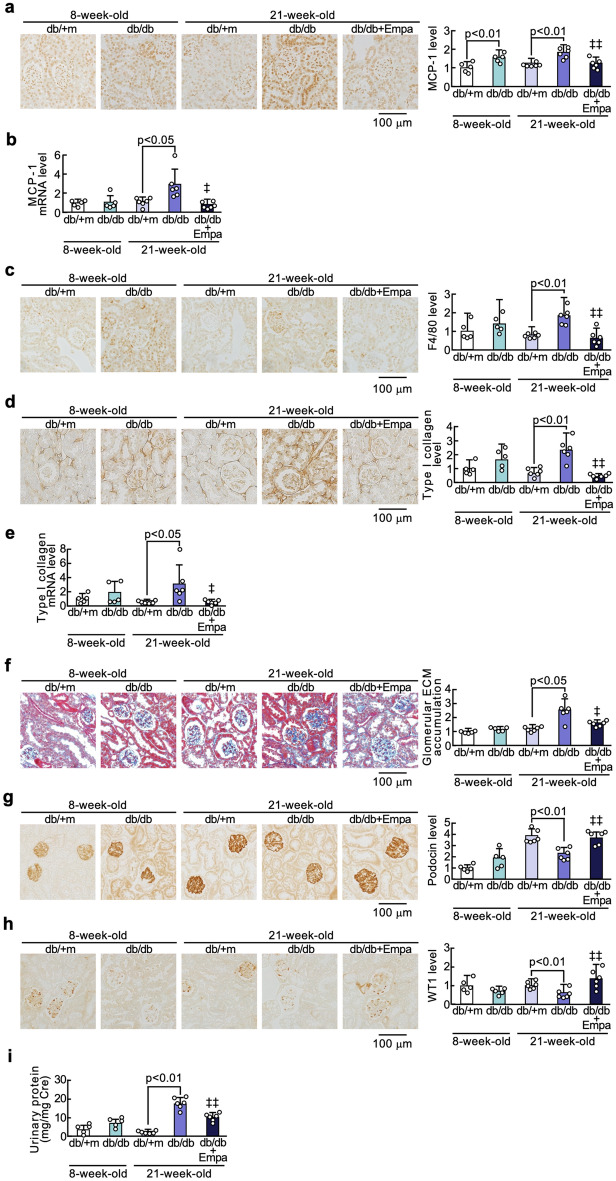

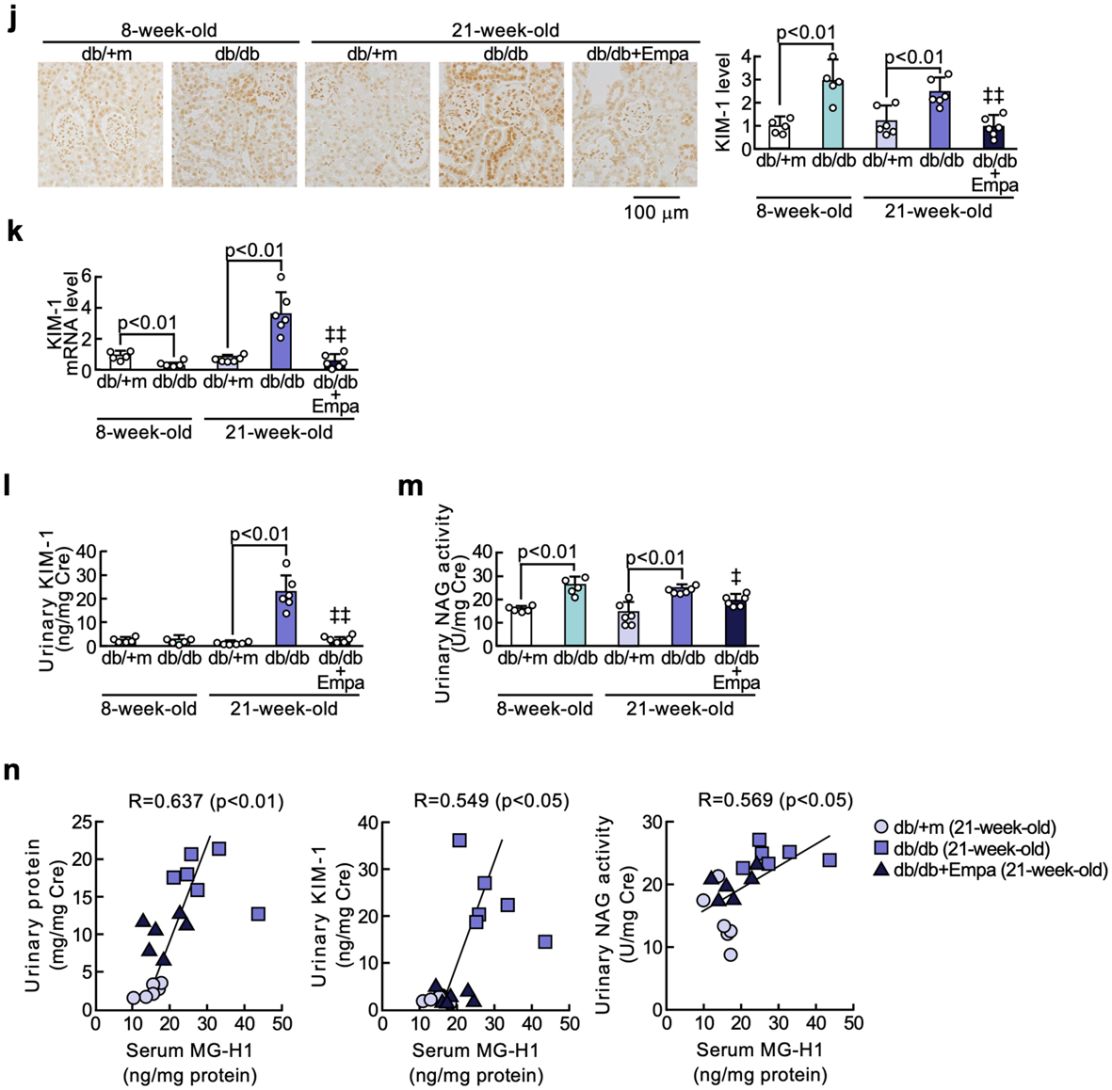
Effects of empagliflozin on renal damage of diabetic mice. Levels of MCP-1 protein (**a**), MCP-1 mRNA (**b**), F4/80 (**c**), type 1 collagen (**d**), type 1 collagen mRNA (**e**), glomerular extracellular matrix accumulation (**f**), podocin (**g**), WT-1 (**h**), KIM-1 (**j**), and KIM-1 mRNA (**k**) in the kidneys, proteinuria (**i**), and urinary KIM-1 excretion (**l**) and NAG activity (**m**)**. a**, **c**, **d**, **f**–**h**, and **j**: Each left panel shows representative immunostainings of MCP-1 protein (**a**), F4/80 (**c**), type 1 collagen (**d**), glomerular extracellular matrix accumulation (**f**), podocin (**g**), WT-1 (**h**), and KIM-1 (**j**). Each right panel shows the quantitative data. **n** Correlation of serum MG-H1 with proteinuria, and urinary KIM-1 excretion and NAG activity. ‡ and ‡‡, p < 0.05 and p < 0.01 compared with 21-week-old diabetic db/db mice, respectively. Cre, creatinine

As shown in Fig. 2, renal MCP-1 mRNA and KIM-1 levels and urinary NAG activity of 8-week-old db/db mice were significantly higher than those of non-diabetic db/m + mice of the same age. However, there were no significant differences in other renal injury markers between the two groups. Gene and protein expression levels of MCP-1 and type 1 collagen and macrophage infiltration in the kidneys of 21-week-old db/db mice were significantly higher than those of non-diabetic control mice of the same age in association with increased glomerular extracellular matrix accumulation, all of which were suppressed by the treatment of empagliflozin for 13 weeks. Furthermore, compared with 21-week-old non-diabetic control mice, immunostaining levels of podocin and WT-1, markers of podocytes (Matsui et al. 2017) were significantly decreased, and renal gene and protein expressions of KIM-1, proteinuria, and urinary KIM-1 excretion and NAG activity were increased in db/db mice of the same age (Fig. 2g–m). Empagliflozin treatment for 13 weeks significantly restored the decrease in podocin and WT-1 expression levels and inhibited the increase in gene and protein expressions of KIM-1 in the kidneys of 21-week-old db/db mice in association with the reduction in proteinuria, and urinary KIM-1 excretion and NAG activity, all of which urinary markers were significantly correlated with serum levels of MG-H1 (Fig. 2n).

### Effects of empagliflozin on AGE-RAGE-oxidative stress axis in the adipose tissues of diabetic mice

**Fig. 3:**
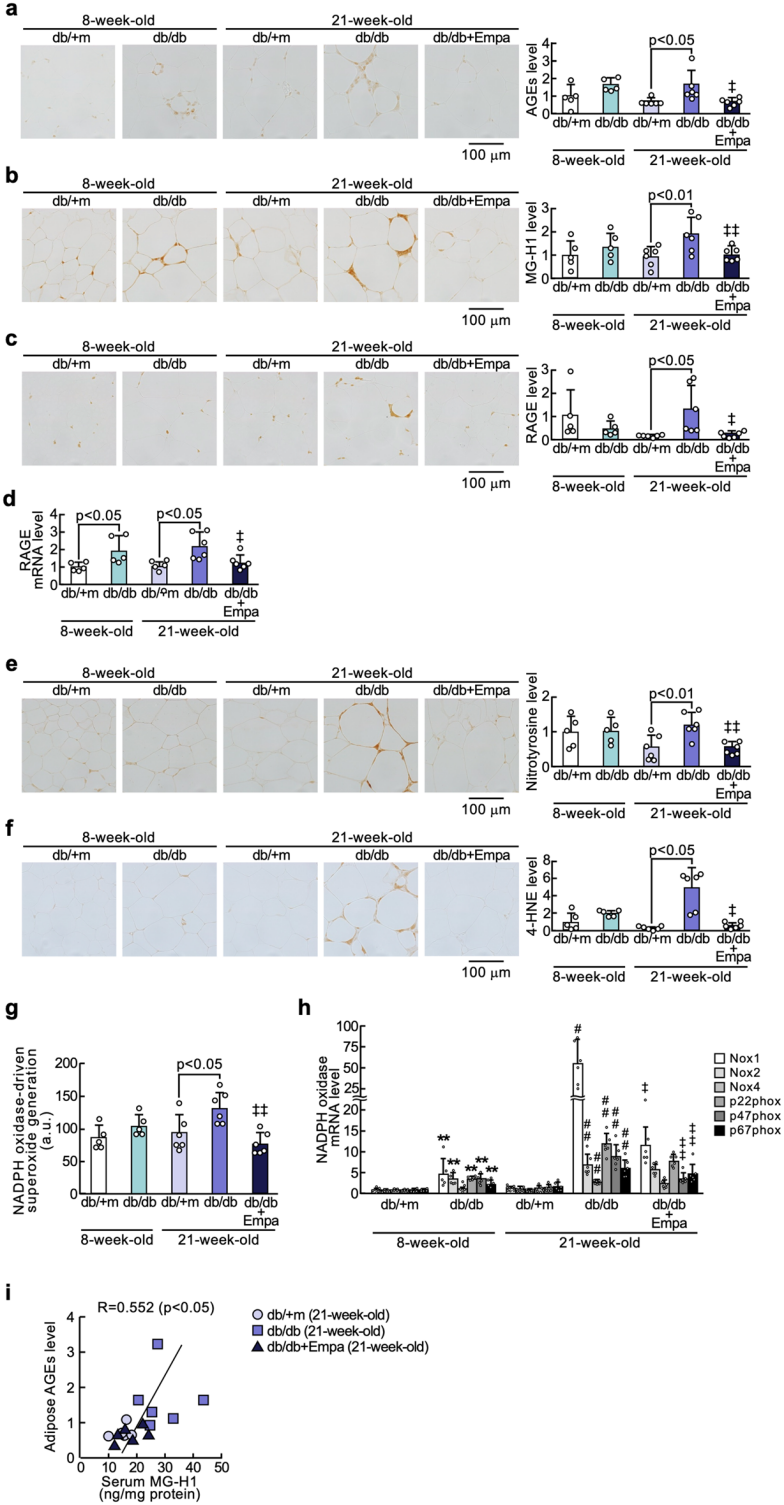
Effects of empagliflozin on AGE-RAGE-oxidative stress axis in the adipose tissues of diabetic mice. Levels of AGEs (**a**), MG-H1 (**b**), RAGE protein (**c**), RAGE mRNA (**d**), nitrotyrosine (**e**), 4-HNE (**f**), NADPH oxidase-derived superoxide generation (**g**), and gene expression levels of components of NADPH oxidase (**h**). **a**–**c**, **e**, and **f**: Each left panel shows representative immunostainings of AGEs (**a**), MG-H1 (**b**), RAGE protein (**c**), nitrotyrosine (**e**), and 4-HNE (**f**) in the adipose tissues. Each right panel shows the quantitative data. **i**: Correlation of serum MG-H1 with adipose tissue AGEs levels. **, p < 0.01 compared with 8-week-old non-diabetic db/m + mice. # and ##, p < 0.05 and p < 0.01 compared with 21-week-old non-diabetic db/m + mice, respectively. ‡ and ‡‡, p < 0.05 and p < 0.01 compared with 21-week-old diabetic db/db mice, respectively

As shown in Fig. 3a–g, AGEs, MG-H1, RAGE mRNA and protein levels, nitrotyrosine, 4-HNE, a marker of lipid peroxidation, and NADPH oxidase-derived superoxide generation in the adipose tissues of 21-week-old diabetic mice were significantly higher than those of non-diabetic control mice of the same age, all of which were suppressed by the treatment with empagliflozin for 13 weeks. Adipose tissue gene expression levels of components of NADPH oxidase except for *Nox4* in 8-week-old db/db mice were significantly elevated in diabetic db/db mice at 8 and 21 weeks old compared with non-diabetic db/m + mice of the same ages, respectively (Fig. 3h). Empagliflozin treatment for 13 weeks significantly inhibited the increase in gene expression levels of *Nox1*, *p47phox* and *p67phox* in the adipose tissues of diabetic mice at 21 weeks old. Serum MG-H1 levels of mice at 21 weeks of age were correlated with AGEs levels in the adipose tissues (Fig. 3i).

### Effects of empagliflozin on adipocytokine expressions in the adipose tissues of diabetic mice

**Fig. 4:**
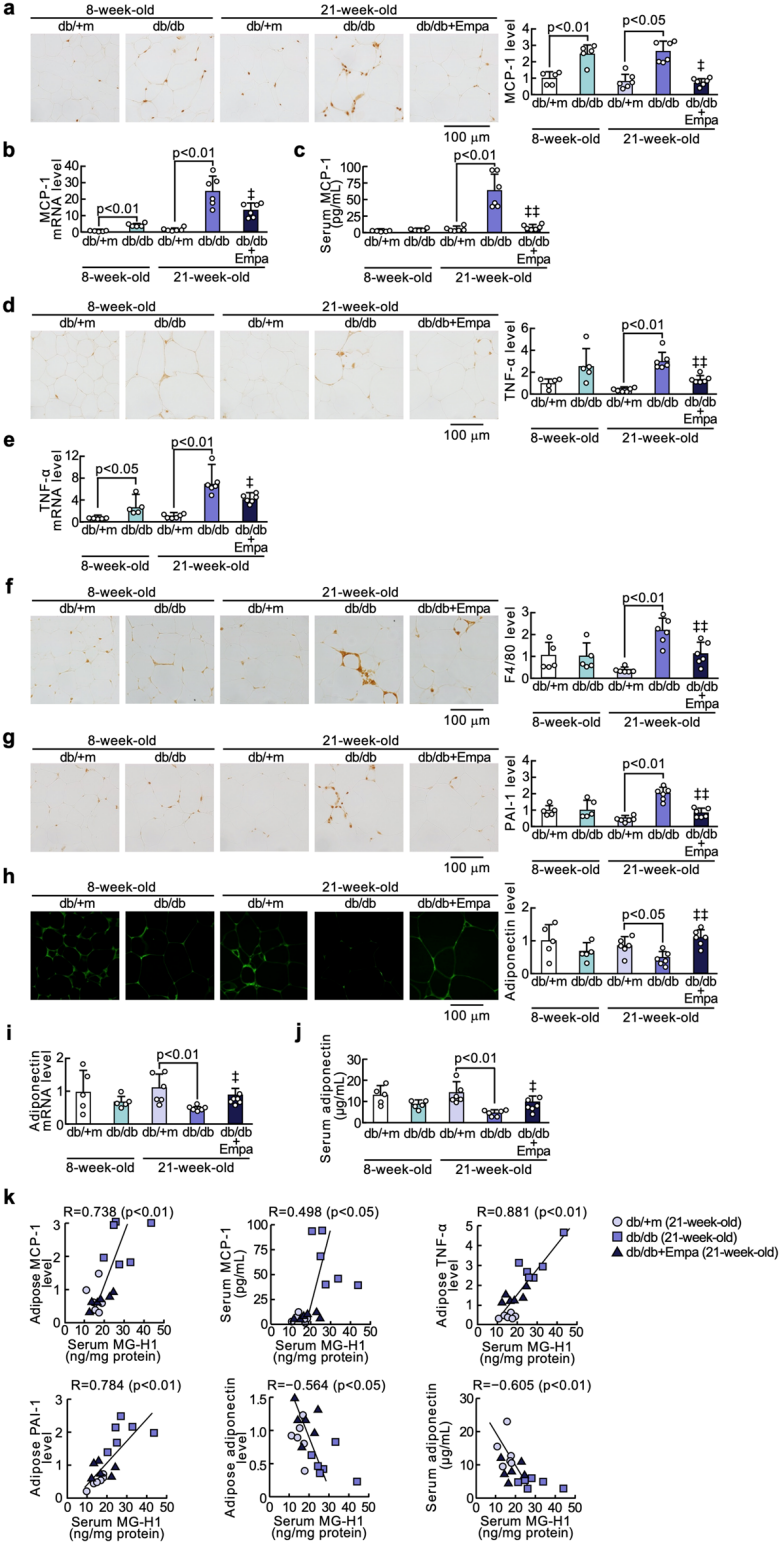
Effects of empagliflozin on adipocytokine expressions in the adipose tissues of diabetic mice. Levels of MCP-1 protein (**a**), MCP-1 mRNA (**b**), TNF-α protein (**d**), TNF-α mRNA (**e**), F4/80 (**f**), PAI-1 (**g**), adiponectin protein (**h**), and adiponectin mRNA (**i**) in the adipose tissues, and serum levels of MCP-1 (**c**) and adiponectin (**j**). **a**, **d**, and **f**–**h**: Each left panel shows representative immunostainings of MCP-1 protein (**a**), TNF-α (**d**), F4/80 (**f**), PAI-1 (**f**), and adiponectin (**h**). Each right panel shows the quantitative data. **k** Correlation of serum MG-H1 with adipose tissue MCP-1, TNF-α, PAI-1, adiponectin, and serum levels of MCP-1 and adiponectin. ‡ and ‡‡, p < 0.05 and p < 0.01 compared with 21-week-old diabetic db/db mice, respectively

As shown in Fig. 4, although MCP-1 mRNA and protein levels and *Tnf-α* gene expression in the adipose tissues of 8-week-old db/db mice were significantly higher than those of non-diabetic mice of the same age, the other adipocytokine levels did not differ between the two groups. Adipose tissue gene and protein expressions of MCP-1 and TNF-α and PAI-1 levels, macrophage infiltration, and serum MCP-1 values were significantly increased in 21-week-old db/db mice compared with non-diabetic mice at 21 weeks old, all of which were inhibited by the treatment with empagliflozin for 13 weeks (Fig. 4a–g). Empagliflozin treatment also significantly restored the decrease in gene and protein expression levels of adiponectin in the adipose tissues as well as serum adiponectin values of 21-week-old db/db mice (Fig. 4h). Serum levels of MG-H1 of mice at 21 weeks old were significantly associated with adipose tissue levels of MCP-1, TNF-α, PAI-1, and adiponectin (inversely) and serum levels of MCP-1 and adiponectin (inversely) (Fig. 4k).

## Discussion

The salient findings of the present studies are (1) empagliflozin treatment for 13 weeks significantly inhibited the increases in AGEs, MG-H1, RAGE, nitrotyrosine, gene expression levels of *Nox1* and *p47phox*, components of NADPH oxidase and its derived ROS generation in the kidneys of 21-week-old db/db type 2 diabetic mice, (2) inflammatory and fibrotic reactions, including MCP-1 expression, macrophage infiltration, type 1 collagen expression, and extracellular matrix accumulation in the kidneys were increased, whereas podocyte markers were decreased in db/db mice at 21 weeks old compared with non-diabetic mice of the same age, all of which were ameliorated by the treatment with empagliflozin for 13 weeks in association with the reductions in proteinuria, and urinary KIM-1 excretion and NAG activity, (3) compared with non-diabetic mice at 21 weeks old, AGEs, MG-H1, RAGE, gene expression levels of component of NADPH oxidase, ROS, and oxidative stress markers in the adipose tissues were also increased in db/db mice of the same age, which were associated with the increases in inflammatory and thrombogenic markers as well as the decrease in adiponectin expression, (4) empagliflozin treatment improved all of these pathological changes observed in the adipose tissues of db/db mice, and (5) serum levels of MG-H1 of 21-week-old mice were significantly associated with renal and adipose tissue AGEs, proteinuria, urinary KIM-1 excretion and NAG activity, adipose tissue inflammatory and fibrotic markers, and serum levels of MCP-1 and adiponectin (inversely). These observations suggest that empagliflozin, a SGLT2i could ameliorate renal and metabolic derangements in obese type 2 diabetic mice partly by blocking the AGE-RAGE-ROS axis in the kidneys and adipose tissues.

A growing body of evidence from in vitro-, animal, and clinical observational studies have suggested the pathological involvement of AGE-RAGE-ROS axis in the development and progression of diabetic kidney disease (Matsui et al. 2017; Yamagishi and Matsui 2013; Tsuchida et al. 1999; Wendt et al. 2003; Flyvbjerg et al. 2004; Kaida et al. 2013; Yamagishi and Imaizumi 2005; Reiniger et al. 2010; Grosjean et al. 2018; Yamamoto et al. 2010; Adeshara et al. 2022; Semba et al. 2010, 2009; Yubero-Serrano et al. 2015). Indeed, we, along with others, have shown that the blockade of the AGE-RAGE-ROS axis by inhibitors of AGEs formation, exogenously administered soluble form of RAGE, neutralizing RAGE antibodies, DNA-aptamers raised against AGEs or RAGE, or gene deletion of RAGE could inhibit inflammatory and fibrotic reactions in the diabetic kidneys with preservation of podocyte loss and reduction of albuminuria (Matsui et al. 2017; Yamagishi and Matsui 2013; Tsuchida et al. 1999; Wendt et al. 2003; Flyvbjerg et al. 2004; Kaida et al. 2013; Yamagishi and Imaizumi 2005; Reiniger et al. 2010; Grosjean et al. 2018). Progressive deterioration of albuminuria, mesangial expansion and glomerulosclerosis were observed in diabetic RAGE transgenic mice compared with diabetic control mice (Yamamoto et al. 2010). Moreover, circulating levels of AGEs and tissue expression of AGEs and RAGE were positively associated with oxidative stress and inflammatory biomarkers in type 2 diabetes patients with nephropathy (Adeshara et al. 2022). Population-based studies of community-dwelling adults revealed that circulating levels of AGEs were independently associated with chronic kidney disease and could predict the decline in renal function (Semba et al. 2010, 2009). In addition, a randomized clinical study with sevelamer carbonate has shown that it reduces circulating and cellular AGEs, oxidative stress and inflammatory markers in type 2 diabetes patients with diabetic kidney disease, and resultantly decreases albuminuria in the subgroups aged < 65 years (Yubero-Serrano et al. 2015). These findings suggest that suppression of the AGE-RAGE-ROS axis in the kidneys by empagliflozin may contribute to nephroprotection in obese and insulin resistant db/db mice.

In the present study, empagliflozin treatment not only decreased fasting blood glucose, but also reduced HOMA-IR and a marker of systemic inflammation, CRP in db/db mice. Besides hyperglycemia, oxidative stress, chronic inflammation, and insulin resistance have been known to promote the formation of AGEs, while AGE-RAGE-evoked NADPH oxidase-mediated ROS generation further enhances RAGE expression in various tissues (Kaida et al. 2013; Yamagishi and Imaizumi 2005; Yamagishi and Matsui 2018; Ide et al. 2010; Tan et al. 2010; Tahara et al. 2010, 2012; Yoshida et al. 2006). Moreover, AGEs induce ROS generation and CRP expression via the interaction with RAGE in liver cells (Yoshida et al. 2006 , 2006). These observations may indicate a positive feedback loop among AGE-RAGE-derived ROS generation, AGE accumulation, RAGE expression, and inflammatory reactions in the diabetic kidneys. Empagliflozin could exert beneficial effects on diabeic nephropathy partly by blocking the pathological crosstalk between AGE-RAGE-ROS axis and inflammatory reactions in the kidneys of db/db mice. We have previously reported that replacement of dipeptidyl peptidase-4 inhibitors by tofogliflozin, a SGLT2i reduced circulating levels of AGEs, fasting levels of insulin, white blood cell count, and arterial stiffness in type 2 diabetes patients, although HbA1c levels were modestly, but significantly increased *rather than* decreased following the switching to SGLT2i (Bekki et al. 2019). The findings suggest the glucose-lowering independent, anti-inflammatory, and insulin-sensitizing effects of empagliflozin may partly contribute to the inhibition of AGE-RAGE axis in the diabetic kidneys. Moreover, we found here that E/e' ratio, a marker of diastolic function and cardiac stiffness was ameliorated by empagliflozin treatment in 21-week-old db/db diabetic mice, which result was consistent with the previous report showing that empagliflozin ameliorated diastolic function and left ventricular stiffness in non-diabetic patients with heart failure (Santos-Gallego et al. 2021). Therefore, the improvement of cardiac stiffness by empagliflozin observed here may also be ascribed in part to its inhibitory actions on AGE-RAGE axis.

We have previously found that (1) SGLT2-mediated glucose uptake to proximal tubular cells could induce oxidative stress, apoptosis, inflammatory reactions, and RAGE expression, thus being involved in tubulointerstitial injury, (2) empagliflozin treatment inhibits inflammatory and fibrotic reactions in the kidneys of type 1 diabetic rats by inhibiting the AGE-RAGE axis, and subsequently reduces urinary excretions of 8-hydroxydeoxyguanosine and l-fatty acid binding protein (l-FABP), markers of oxidative stress and tubular injury, respectively (Maeda et al. 2013; Ojima et al. 2015). These previous studies suggest that empagliflozin may protect against the AGE-RAGE-elicited tubulointerstitial damage in the kidneys of type 1 diabetic rats. However, the use of empagliflozin is not approved for the treatment of type 1 diabetic patients. Moreover, we have previously reported that tofogliflozin inhibits renal damage in KKAy/Ta mice, obese and type 2 diabetic animals (Ishibashi et al. 2016 , 2016), but the effects of SGLT2is on the AGE-RAGE axis in the kidneys of type 2 diabetic animals remain unclear. These are reasons why we examined here whether empagliflozin could ameliorate renal damage in db/db mice, obese and insulin resistant type 2 diabetes animals. Given the finding that tofogliflozin lowers albuminuria in type 2 diabetes patients, 40% of which effect could be attributable to its beneficial action on tubulointerstitial damage, as evidenced by decrease of urinary l-FABP (Yanagisawa et al. 2020), our present study suggests that empagliflozin may reduce proteinuria in db/db mice partly via attenuation of the tubulointerstitial injury caused by the AGE-RAGE-ROS axis.

In this study, we found that serum levels of MG-H1 were correlated with kidney AGEs levels, proteinuria, and urinary KIM-1 excretion and NAG activity in 21-week-old mice. Among various types of AGEs, circulating levels of MG-H1 adduct was reported to be strongly correlated with the severity of chronic kidney disease in both type 2 diabetes and non-diabetic patients (Rabbani et al. 2022). Furthermore, circulating levels of MG-H1 levels were associated with incident macroalbuminuria and high risk of chronic kidney disease in type 2 diabetes participants of Action to Control Cardiovascular Risk in Diabetes (ACCORD) and Veterans Affairs Diabetes Trial (VADT) (Koska et al. 2022). Therefore, the present findings and previous observations suggest that circulating levels of MG-H1 levels could be a biomarker of diabetic kidney disease, which may be one of the molecular targets of empagliflozin. In support of this speculation, we have previously found that (1) serum levels of glyceraldehyde-derived AGEs are elevated under diabetic and oxidative stress conditions, and correlated with inflammatory and thrombotic biomarkers, insulin resistance, and vascular inflammation in both type 2 diabetes and non-diabetic individuals (Yamagishi et al. 2015), (2) MG-H1, one of the AGEs formed from glyceraldehyde, elicits ROS generation and inflammatory reactions in endothelial cells via interaction with RAGE (Ishibashi et al. 2017), and (3) circulating MG-H1 levels are correlated with asymmetric dimethylarginine, an endogenous inhibitor of nitric oxide synthase, which is also a marker of cardiorenal disease and insulin resistance (Tahara et al. 2019; Ueda et al. 2014).

In the present study, we found that empagliflozin treatment inhibited AGEs, RAGE, NADPH oxidase-derived ROS generation, and inflammatory reactions in the adipose tissues of db/db mice in association with restoration of adiponectin expression levels and improvement of insulin resistance. Furthermore, at 21-week-old mice, serum levels of MG-H1 were significantly associated with increased inflammatory markers and decreased adiponectin levels in the adipose tissues and the serum. We have previously shown that AGE-RAGE-induced NADPH oxidase oxidase-mediated ROS generation could contribute to inflammatory reactions and resultantly evoke insulin resistance in the adipose tissues in association with reduction of adiponectin in fructose-fed rats (Kaseda et al. 2020). Therefore, the present study has further supported the concept that AGE-RAGE-ROS axis in the adipose tissues could be a therapeutic target for ameliorating impairment of adipocytokine profiles and insulin resistance in type 2 diabetes. Given that serum levels of glyceraldehyde-derived AGEs, which could include MG-H1, are associated with insulin resistance and decreased adiponectin levels (Hyogo et al. 2007), circulating MG-H1 levels may also be a novel biomarker of adipose tissue inflammation, disturbed adipocytokine expressions, and insulin resistance in type 2 diabetes.

Adiponectin has been reported to have nephroprotective properties in diabetes; it could reduce albuminuria, glomerular hypertrophy, and renal inflammatory reactions in diabetes (Zha et al. 2017). Moreover, we have previously found that blocking the binding of AGEs to RAGE by DNA-aptamer raised against RAGE not only reduces renal levels of AGEs, RAGE, and NADPH oxidase-derived ROS, albuminuria, and urinary NAG activity with amelioration of histological alterations in glomerular and interstitial areas, but also improves insulin sensitivity and increase adiponectin levels in KKAy/Ta mice (Sotokawauchi et al. 2021). These observations suggest the pathophysiological connection of metabolic derangements to renal injury in obese and insulin resistant type 2 diabetes, which may be partly mediated by the activation of AGE-RAGE axis.

There are several limitations in the present study. First, in this study, empagliflozin significantly decreased fasting blood glucose and HOMA-IR compared to diabetic control mice. Nevertheless, db/db + Empa mice showed a higher body weight and a lipid profile that was not ameliorated in respect to db/db mice. Food intake was significantly greater in empagliflozin-treated diabetic mice than non-treated diabetic mice of the same age, which may partly explain these unexpected results. We could not examine the effects of empagliflozin on fat mass/visceral obesity, but there were no significant differences of adipocyte size between empagliflozin-treated and non-treated db/db mice at 21 weeks old (data not shown). Second, while F4/80 is a surface receptor and MCP-1 is a chemokine, both of which should have a cytoplasmic staining, some positive spots in immunohistochemistry seem to be localized to nuclei. However, the maximum and minimum sizes of positive spots were 1 µm and 8 µm, respectively, which were not necessarily consistent with an average nuclear diameter of 6 µm for mouse cells. Since it is difficult to differentiate certain cellular components, such as nucleus and cytoplasm by immunohistochemistry, it would be helpful to perform immunofluorescent stainings for these inflammatory markers to reduce some problem of background signal due to DAB staining. In this study, due to lack of frozen samples, we could not perform additional experiments. However, we confirmed here that inflammatory signals, including renal and adipose tissue MCP-1 mRNA levels and serum levels of MCP-1, were significantly inhibited by the treatment of empagliflozin in 21-week-old db/db mice. Third, we found here that empagliflozin treatment caused a significant reduction in proteinuria of db/db mice despite a worsening in the blood urea nitrogen and no effects on kidney hypertrophy. Elevation of hematocrit by empagliflozin treatment may partly explain the worsening in blood urea nitrogen levels. Empagliflozin may reduce proteinuria in part via improvement of podocyte injury. Fourth, in the present study, empagliflozin treatment significantly decreased fasting blood glucose in 21-week-old db/db diabetic mice. Therefore, glucose-lowering effects of empagliflozin may also contribute to the suppression of AGE-RAGE axis in our model. Fifth, effects of empagliflozin may differ among tissues in db/db diabetic mice. In the hearts, the beneficial effects of empagliflozin may also occur independent of oxidative stress and changes in AGE-RAGE signaling. In addition, effects of empagliflozin on vascular remodeling may also contribute to nephroprotection in db/db mice. Sixth, although we did not know the exact molecular mechanism of increased NADPH oxidase by AGE-RAGE axis, interaction of AGEs with RAGE may increase NADPH oxidase activity by up-regulation of mRNA levels of its membrane and cytosolic components partly via oxidative stress generation.

In conclusion, our present findings suggest that empagliflozin could ameliorate renal and metabolic derangements in obese and insulin resistant type 2 diabetes animals partly by attenuating the AGE-RAGE-ROS axis in the kidneys and adipose tissues. Serum MG-H1 levels may be a biomarker of renal and metabolic derangements in type 2 diabetes.

## Data Availability

The datasets used and analyzed during the current study are available from the corresponding author on reasonable request.

## Abbreviations

4-HNE: 4-Hydroxynonenal
ALT: Alanine aminotransferase
AST: Aspartate aminotransferase
CRP: C-reactive protein
Empa: Empagliflozin
KIM-1: Kidney injury molecular-1
l-FABP: L-fatty acid binding protein
MCP-1: Monocyte chemoattractant protein-1
MG-H1: *N*^ε^-(5-Hydro-5-methyl-4-imidazolon-2-yl)-ornithine
NAG: *N*-Acetyl-β-d-glucosaminidase
PAI-1: Plasminogen activator inhibitor-1
RAGE: Receptor for AGEs
ROS: Reactive oxygen species
SGLT2: Sodium–glucose cotransporter 2
SGLT2i: SGLT2 inhibitor(s)
TNF-α: Tumor necrosis factor-α
WT1: Wilms’ tumor 1
